# RETRACTED: Hu et al. Classification and Recognition of Building Appearance Based on Optimized Gradient-Boosted Decision Tree Algorithm. *Sensors* 2023, *23*, 5353

**DOI:** 10.3390/s26195988

**Published:** 2026-09-22

**Authors:** Mengting Hu, Lingxiang Guo, Jing Liu, Yuxuan Song

**Affiliations:** 1 School of Civil Engineering and Architecture, Xiamen University of Technology, Xiamen 361024, China; 2 The Arts and Design College Xiamen, Fuzhou University, Xiamen 361000, China; 3 College of Forestry and Grassland, Jilin Agriculture University, Changchun 130118, China

The journal retracts the article titled “Classification and Recognition of Building Appearance Based on Optimized Gradient-Boosted Decision Tree Algorithm” [1], cited above.

Following publication, the authors notified the Editorial Office of errors in the experimental data presented in this article [1].

In accordance with the journal’s standard procedures, the Editorial Office and Editorial Board conducted an investigation that confirmed major flaws in the methodology and experimental results. The authors cooperated with the Editorial Office and Editorial Board throughout the investigation and submitted revised material. However, after careful evaluation, the Editorial Board determined that the extent of the required changes was too substantial to be addressed through a correction.

Given the impact of these errors on the validity and the reliability of the study’s findings, the authors and the Editorial Board agreed to retract this article as per MDPI’s retraction policy.

This retraction was approved by the Editor-in-Chief of the *Sensors* journal.
